# Editorial: Calcium and parathormone: an update on the clinical presentation and new therapies

**DOI:** 10.3389/fendo.2023.1199056

**Published:** 2023-04-28

**Authors:** Mara Carsote, Mihaela Popescu, Alice Elena Ghenea, Mihaela Jana Tuculina, Ana Valea

**Affiliations:** ^1^ Department of Endocrinology, Carol Davila University of Medicine and Pharmacy, Bucharest, Romania; ^2^ Department of Endocrinology, C.I. Parhon National Institute of Endocrinology, Bucharest, Romania; ^3^ Department of Endocrinology, University of Medicine and Pharmacy of Craiova, Craiova, Romania; ^4^ Department of Bacteriology–Virology–Parasitology, University of Medicine and Pharmacy of Craiova, Craiova, Romania; ^5^ Department of Endodontics, University of Medicine and Pharmacy of Craiova, Craiova, Romania; ^6^ Department of Endocrinology, Iuliu Hatieganu University of Medicine and Pharmacy, Cluj-Napoca, Romania; ^7^ Department of Endocrinology, Clinical County Hospital, Cluj-Napoca, Romania

**Keywords:** calcium, parathyroid hormone (PTH), parathyroidectomy (PTX), primary hyperparathyroidism, hypoparathyroidism, thyroidectomy, thyroid cancer, cognition

## Introduction

**Figure 1:**
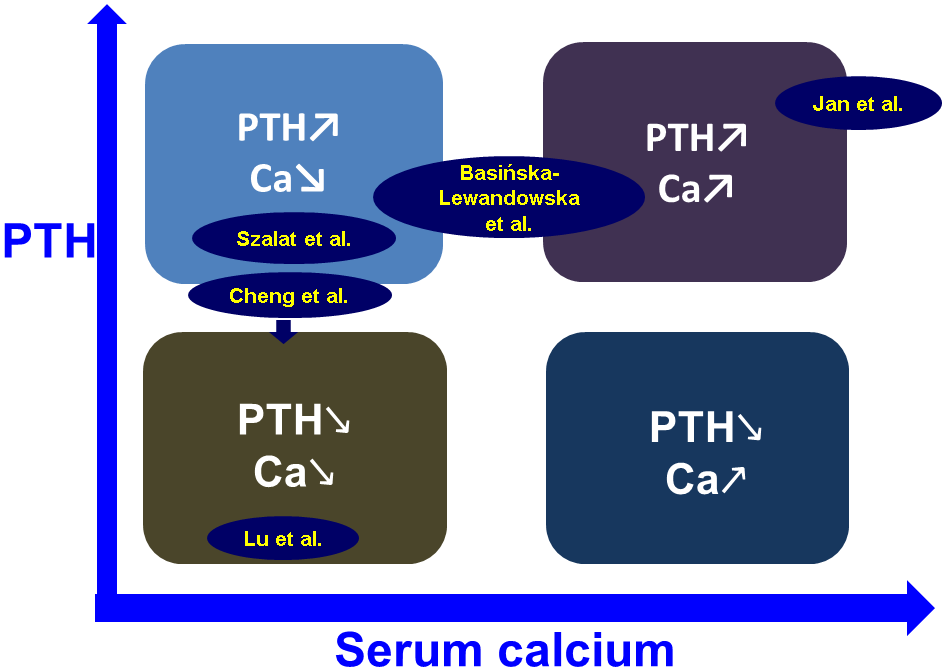
Placing the topics of the five papers published in this Research Topic in the spectrum field of calcium and PTH – associated conditions (see links in text).

The current Research Topic entitled “*Calcium and Parathormone: An Update on the Clinical Presentation and New Therapies*” covers a wide range of multidisciplinary disorders involving various combinations of high or low calcium and parathormone levels. (**Figure 1**) The five published articles represent the contributions of 28 authors from four countries on two continents (Poland, Israel, China, and Taiwan). We consider their work to be a valuable and impactful contribution to the field of parathyroid-associated diseases.

## PTH anomalies as contributors to ocular (corneal) abnormalities

Band keratopathy, a degenerative disease caused by calcium hydroxyapatite deposition in the superficial layers of the cornea, has been commonly reported in association with various causes, including endocrine conditions such as chronically abnormal blood glucose or calcium and phosphorus levels (1). Jan et al. published the largest population-based, case-control study concerning this ophthalmic condition and its correlation with different comorbidities and socio-demographic factors. The sample size was more than 2500 subjects diagnosed with this ocular anomaly, and six controls were selected for each patient. A statistically significant higher prevalence was observed in individuals diagnosed with hyperparathyroidism (primary, secondary, or tertiary, including renal type), diabetes mellitus, or chronic kidney disease. Moreover, this eye disorder was associated with age, geographic region of origin, and occupation. Abnormal PTH levels are significant findings related to this corneal condition, with the hormonal component playing a crucial role in its pathogenesis.

## Normocalcemic primary hyperparathyroidism

This new phenotype of parathyroid disease with elevated PTH and normal calcium concentrations remains controversial in terms of defining criteria, natural evolution, and specific approach protocols. Essentially, the condition should be distinguished from hypovitaminosis D – associated with secondary hyperparathyroidism (2). Basińska-Lewandowska et al. evaluated the disease with respect to the (bi-annual) seasonal pattern of vitamin D variations (without supplementation) according to screening assessments among primary care controls. Vitamin D deficiency affected up to two-thirds of the population studied in the spring, compared with one-third in the fall. This may explain why high PTH and normal calcium are more likely to be caused by secondary hyperparathyroidism in the spring and normocalcemic primary hyperparathyroidism in the fall. This is a significant aspect to be taken into consideration by primary care physicians when providing vitamin D supplementation and referring patients for endocrine evaluation.

## Neurocognitive impairment in primary hyperparathyroidism

Quality of life, including neurocognitive dysfunction, represents an ongoing controversy in primary hyperparathyroidism, as it is not yet considered an indication for parathyroidectomy (3). Szalat et al. assessed these characteristics before and 6 months after parathyroid surgery in a prospective, case-control study. The authors showed that postoperative functions such as long-term auditory memory, visual memory, visual attention, and concentration skills were statistically significantly improved, in contrast to frontal lobe–related abilities. No elements of depression were identified prior to parathyroidectomy. The authors also reviewed the English literature and found 16 prospective studies analyzing the neurocognitive features in relation to primary hyperparathyroidism (as a pre- and post-operative panel) with heterogeneous results. These sets of data suggest that neurocognitive function-related anomalies are still an open question in terms of routine use in primary hyperparathyroidism and the clinical decision-making process for parathyroidectomy.

## Renal hyperparathyroidism

End-stage renal disease is associated with numerous multidisciplinary complications; while hyperparathyroidism worsens the overall skeletal, cardiovascular, and quality-of-life–associated picture, the development of post-parathyroidectomy hypocalcemia may add to the burden of comorbidities and mortality (4). Cheng et al. provided a predictive model based on baseline levels of PTH, calcium, and the bone turnover marker alkaline phosphatase to anticipate postoperative hypocalcemia in patients with renal hyperparathyroidism. They conducted a randomized control study, which concluded that when the risk of hypocalcemia (as calculated by the model) is above the 66.9% cutoff, active and immediate intervention is required, with a clear benefit to patients.

## Post-thyroidectomy hypoparathyroidism

Calcium and PTH abnormalities after thyroidectomy, especially in patients with thyroid cancer, are the most frequent complications, especially in cases with low preoperative 25-hydroxyvitamin D levels and extensive surgery (5). In a retrospective cohort study, Lu et al. included more than 500 patients who underwent total thyroidectomy in addition to parathyroid auto-transplantation. The 6-month postoperative analysis showed that two-thirds of the subjects developed hypoparathyroidism, a complication that correlated with the type of procedure (open surgery), an increased number of transplanted parathyroid glands, and, interestingly, with fasting glycemia and positive anti-thyroid antibodies consistent with a diagnosis of Hashimoto’s thyroiditis. These parameters may help clinicians promptly provide medical therapy in these cases.

## Conclusion

The complex field of calcium and parathormone–derived disorders is still challenging; we consider this Research Topic to add value to our current knowledge, and we were honored to host it. We thank all the authors for their contributions.

## Author contributions

MC wrote the draft and AV provided the final editing of the paper; MP, AG, and MT read and commented the article. All authors contributed to the article and approved the submitted version.

## References

[1] Baser H Cuhaci N Topaloglu O Yulek F Ugurlu N Ersoy R. Is there any association between primary hyperparathyroidism and ocular changes, such as central corneal thickness, retinal thickness, and intraocular pressure? Endocrine (2016) 51(3):545–50. doi: 10.1007/s12020-015-0724-5 26318316

[2] Cusano NE Cetani F. Normocalcemic primary hyperparathyroidism. Arch Endocrinol Metab (2022) 66(5):666–77. doi: 10.20945/2359-3997000000556 PMC10118830 36382756

[3] Cipriani C Cianferotti L. Quality of life in primary hyperparathyroidism. Endocrinol Metab Clin North Am (2022) 51(4):837–52. doi: 10.1016/j.ecl.2022.04.007 36244696

[4] Bilezikian JP Khan AA Silverberg SJ Fuleihan GE Marcocci C Minisola S. Evaluation and management of primary hyperparathyroidism: Summary statement and guidelines from the fifth international workshop. J Bone Miner Res (2022) 37(11):2293–314. doi: 10.1002/jbmr.4677 36245251

[5] Yan XQ Zhang ZZ Yu WJ Ma ZS Chen ML Xie BJ. Prophylactic central neck dissection for cN1b papillary thyroid carcinoma: a systematic review and meta-analysis. Front Oncol (2022) 11:803986. doi: 10.3389/fonc.2021.803986 35096606 PMC8795744

